# Assessment of 5-HT_7_ Receptor Agonists Selectivity Using Nociceptive and Thermoregulation Tests in Knockout versus Wild-Type Mice

**DOI:** 10.1155/2012/312041

**Published:** 2012-06-19

**Authors:** Alex Brenchat, Maria Rocasalbas, Daniel Zamanillo, Michel Hamon, José Miguel Vela, Luz Romero

**Affiliations:** ^1^Department of Pharmacology, Drug Discovery and Preclinical Development, ESTEVE, Avenida Mare de Déu de Montserrat 221, 08041 Barcelona, Spain; ^2^UMR 894 INSERM-CPN/UPMC, Faculté de Médecine Pierre et Marie Curie, Site Pitié-Salpêtrière, 91 boulevard de l'Hôpital, 75634 Paris Cedex 13, France

## Abstract

No study has ever examined the effect of 5-HT_7_ receptor agonists on nociception by using 5-HT_7_ receptor knockout mice. Basal sensitivity to noxious heat stimuli and formalin-induced nociception in both phase I and II of the formalin test did not differ in 5-HT_7_ receptor knockout mice and paired wild-type controls. Similarly, there was no significant difference in basal body temperature between both genotypes. Subcutaneous administration of 5-HT_7_ receptor agonists AS-19 (10 mg/kg), E-57431 (10 mg/kg), and E-55888 (20 mg/kg) significantly reduced formalin-induced licking/biting behavior during the phase II of the test in wild-type but not in 5-HT_7_ receptor knockout mice. At these active analgesic doses, none of the three 5-HT_7_ receptor agonists modified the basal body temperature neither in wild-type nor in 5-HT_7_ receptor knockout mice. However, a significant decrease in body temperature was observed at a higher dose (20 mg/kg) of AS-19 and E-57431 in both genotypes. Our data strongly suggest that the 5-HT_7_ receptor agonists AS-19, E-57431, and E-55888 produce antinociception in the formalin test by activating 5-HT_7_ receptors. These results also strengthen the idea that the 5-HT_7_ receptor plays a role in thermoregulation, but by acting in concert with other receptors.

## 1. Introduction

The 5-HT_7_ receptor has been cloned from different genomes and its binding profile is consistent across species and between cloned and native receptors [1, 2]. In recent years, considerable efforts have focused on the development of selective 5-HT_7_ receptor agonists and antagonists. To date, the search for 5-HT_7_ receptor antagonists has led to the discovery of LY215840 [3], SB-258719 [4], DR4004 [5], SB-269970 [6], and SB-656104-A [7]. Regarding 5-HT_7_ receptor agonists, AS-19 [8, 9], MSD-5a [10], LP-44 [11], LP-211 [12], E-55888 [13], and E-57431 [14] have been developed. However, most of these agonists display rather modest selectivity because their affinity for the 5-HT_7_ type is only 11-fold higher than for 5-HT_1D_ in case of AS-19 [13], 28.6-fold higher than for 5-HT_1A_ in case of MSD-5a [10], and 33-fold higher than for dopamine D2 receptor [15], and 5-14-fold higher than for 5-HT_1B_, 5-HT_2B_, 5-HT_2C_, and 5-HT_5A_ in case of LP-211 [16]. Indeed, among 5-HT_7_ receptor agonists, only E-55888 and E-57431 seem to have a satisfactory selectivity with affinity for the 5-HT_7_ receptor 280-fold higher than for 5-HT_1A_ and 112.7-fold higher than for 5-HT_1D_, respectively [13] (see Table 1). When tested in a functional assay, 5-HT_7_ receptor agonists concentration dependently increased cAMP formation in HEK-293F/h5-HT_7_ cells. AS-19 has been found to behave as a potent (EC_50_ = 9 ± 1 nM) but partial 5-HT_7_ receptor agonist, with a maximal effect reaching 77% of that of 5-HT [13]. However, E-55888 and E-57431 behave as full agonists, with efficacies (*E*_max⁡_ = 99 ± 1% and 94.5 ± 1%, resp.) and potencies (EC_50_ = 16 ± 1 nM and 21.5 ± 1 nM) similar to those of 5-HT, as previously described [13, 14].

From data obtained with these pharmacological tools, it has been claimed that 5-HT_7_ receptors are involved in a number of physiological and pathophysiological phenomena such as nociception and thermoregulation. Data supporting a role for 5-HT_7_ receptors in pain control mostly suggest an antinociceptive effect of 5-HT_7_ receptor activation in the CNS and, in contrast, a pronociceptive effect of 5-HT_7_ receptor activation in the periphery [17–23]. However, an overall antinociceptive effect has been observed following systemic administration of the selective 5-HT_7_ receptor agonists AS-19, E-57431, and E-55888 to rodents suffering from neuropathic pain [13, 14]. 

On the other hand, 5-HT_7_ receptors have been involved in the control of body temperature based on studies using some 5-HT_7_ receptor agonists (5-CT, 8-OH-DPAT, and LP-211), 5-HT_7_ receptor antagonists (SB-258719 and SB-269970) and 5-HT_7_ receptor knockout mice. Activation of 5-HT_7_ receptors has been reported to decrease body temperature in a complex manner, in concert with other serotonergic receptors such as the 5-HT_1A_ receptor and/or nonserotonergic receptors [16, 24–28].

In addition to pharmacological studies using 5-HT_7_ receptor agonists and antagonists, the 5-HT_7_ receptor knockout mice may provide a relevant tool to explore the functions of this receptor, and to assess the specificity of ligands supposed to interact selectively with it. Accordingly, the present study examines the effects of the so-called 5-HT_7_ receptor agonists AS-19, E-57431, and E-55888 on formalin-induced pain behavior and thermoregulation in 5-HT_7_ receptor knockout and paired wild-type mice in order to determine the *in vivo* functional selectivity of these ligands at this specific receptor type.

## 2. Materials and Methods

### 2.1. Animals

Male, 5- to 8-week-old, 5-HT_7_ receptor knockout (5-HT_7_R^−/−^) C57BL/6J mice and their wild-type 5-HT_7_R^+/+^ siblings used in this study were provided by Deltagen (CA, USA). Embryonic stem cells derived from the 129/OlaHsd mouse substrain were used to generate chimeric mice. F1 mice were generated by breeding with C57BL/6 females. F2 homozygous mutant mice were produced by intercrossing F1 heterozygous males and females. Successive mating of heterozygous progeny to the inbred C57BL/6J strain was performed for at least 8 generations before the knockout and wild-type homozygous offsprings were used in the present study. Genotyping was performed by PCR analysis using a protocol described by The Jackson Laboratory (http://jaxmice.jax.org/protocolsdb/f?p=116:2:2420567317716723::NO:2:P2_MASTER_PROTOCOL_ID,P2_JRS_CODE:1854,005769). Animals were housed in groups of five, provided with food and water *ad libitum* and kept in controlled laboratory conditions with ambient temperature maintained at 21 ± 1°C and light in 12 h cycles (on at 07:00 h and off at 19:00 h). Experiments were carried out in a sound-attenuated, air-regulated, experimental room. All experimental procedures and animal husbandry were conducted according to ethical principles for the evaluation of pain in conscious animals [29], and to ethical guidelines of the European Communities Council Directive of November 24, 1986 (86/609/EEC). The experimental work received approval by the Local Ethical Committee. 

### 2.2. Drugs

Formaldehyde (37 wt.% solution) was purchased from Panreac (Spain) and dissolved in physiological saline. Drugs used for treatments were AS-19 (dimethyl-[5-(1,3,5-tri-methyl-1H-pyrazol-4-yl)-1,2,3,4-tetrahydro-naphthalen-2(S)-yl]-amine) [8, 9], E-55888 (dimethyl-{2-[3-(1,3,5-trimethyl-1H-pyrazol-4-yl)-phenyl]-ethyl}-amine dihydrochloride) [13], and E-57431 (2-(2-(dimethylamino)ethyl)-4-(1,3,5-tri-methyl-1H-pyrazol-4-yl)phenol) [14]. AS-19 is a potent selective 5-HT_7_ receptor agonist commercially available from Tocris Bioscience (UK), whereas E-55888 and E-57431 are 5-HT_7_ receptor agonists developed by ESTEVE Laboratories (Barcelona, Spain). All three 5-HT_7_ receptor agonists were synthesized for the purpose of this study at ESTEVE, dissolved in aqueous solutions containing 0.5% (hydroxypropyl) methyl cellulose (Sigma-Aldrich, Spain) and administered in a volume of 5 ml/kg through the subcutaneous (s.c.) route. Doses of drugs (referred to their salt forms) and time of evaluation were selected based on previous studies [13, 14] and on pilot experiments in models used in this study. All treatments were performed under blind conditions in independent groups of mice and behavioral evaluation was done 30 min after drug administration.

### 2.3. Nociceptive Behavioral Tests

#### 2.3.1. Tail Flick Test

Animals were placed in a loose plexiglas restrainer with their tail extruding through a hole to perform the tail flick test as previously described [30]. A photobeam was placed on the tail about 4 cm from the tip. The latency to tail flick response was recorded automatically to the nearest 0.1 s. The intensity of the radiant heat source was adjusted to yield baseline latencies between 3 and 5 s in wild-type mice. A cut-off latency of 10 s was imposed to avoid damage of tail tissues.

#### 2.3.2. Tail Immersion Test

Animals were placed in a loose plexiglas restrainer with their tail extending through a hole in the water bath of the apparatus (Stuart Bibby Sterilin Ltd, Water Baths SWB1D, UK), as previously described [31]. The lower 2/3 of the tail was immersed in hot water maintained at a constant temperature of 52.0 ± 0.5°C. The latency between tail immersion and attempts to remove the tail from the hot water bath was recorded. A cut-off latency of 15 s was imposed to avoid damage of tail tissues.

#### 2.3.3. Hot Plate Test

Animals were placed individually on the surface of the hot plate apparatus (PanLab, LE 7406, Spain) surrounded by a plexiglas cylinder (20 cm in diameter, 25 cm high). The temperature of the surface was maintained at 55.0 ± 0.5°C, according to the method previously described [32]. The time between placement and the occurrence of forepaw licking (FPL), hindpaw licking (HPL), or jump was recorded as response latency. A cut-off latency of 240 s was established to avoid damage of paw tissues. 

#### 2.3.4. Formalin Test

Formalin (20 *μ*L of a 2.5% formalin solution; 0.92% of formaldehyde) was injected into the dorsal surface on the right hind paw, as previously described [33]. The formalin test is a valid and reliable model of nociception with two distinct periods of high licking activity that have different nociceptive mechanisms, an early phase lasting the first 5 min and a late phase lasting from 15 to 45 min after the injection of formalin. Mice were placed on a paper surface surrounded by a plexiglas cylinder (20 × 25 cm) and the time spent licking and biting the injected paw was measured using a chronometer. A time course of the licking/biting behaviors was monitored during 45 minutes after formalin injection to evaluate possible differences between genotypes. Drug effects were quantified at 0–5 min (phase I) and 15–30 min (phase II) after formalin injection, two periods of time in which the formalin-induced licking and biting time was high enough to test antinociceptive effects of drugs.

#### 2.3.5. Rectal Temperature

The body temperature was recorded using a precision thermometer (YSI 4600) equipped with a flexible probe (YSI 402). This probe was lubricated with vaseline and inserted 2 cm into the rectum. Temperature recordings were made 20 s following insertion of the probe, as previously described [27, 28].

### 2.4. Data Analysis

Data are presented as mean values ± S.E.M. Statistical analysis to test significant differences among groups was made using ANOVA followed by Bonferroni's post hoc comparison. Unpaired Student's *t*-test was used to test differences between two groups. The level of significance was set at *P* < 0.05. Data analysis and graphing were done using GraphPad Prism software (version 4.0; GraphPad Software Inc., USA).

## 3. Results

### 3.1. Similar Response to Noxious Thermal Stimuli and Formalin-Induced Nociception in 5-HT_7_ Receptor Knockout and Wild-Type Mice

**Figure 1:**
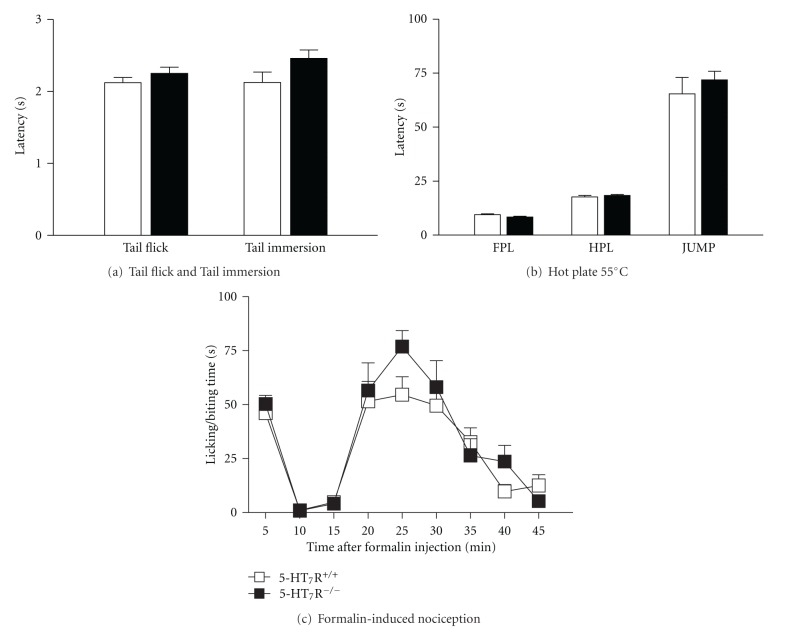
Nociceptive behavior of wild-type and 5-HT_7_ receptor knockout mice in the tail flick and tail immersion tests (a), hot plate 55°C test (b), and formalin test (c). Both genotypes showed similar latency for all measured behaviors in the tail flick, tail immersion, and hot plate tests. Formalin-induced licking and biting of the hind paw injected with formalin in 5-HT_7_ receptor knockout mice did not significantly differ from those in wild-type mice either in phase I (0–5 min) or in phase II (15–45 min). Only a slight but not significant increase of the licking/biting time was observed 25 min after formalin injection in 5-HT_7_ receptor knockout mice compared to wild-type mice. Each bar or symbol represents the mean ± S.E.M. (*n* = 10–12 per group). Forepaw licking: FPL; hindpaw licking: HPL. No significant differences were observed in thermal nociception (unpaired Student's *t*-test) or formalin-induced nociceptive behaviors (Two-Way ANOVA).

Sensitivity to noxious heat measured as the latency of response to thermal stimulation in the tail flick, tail immersion, and hot plate tests was similar in 5-HT_7_ receptor knockout mice and paired wild-type mice (Figure 1). No significant differences between the two genotypes were found in tail withdrawal latency in the tail flick (*t*_49_ = 1.09, *P* = 0.28) and tail immersion (*t*_49_ = 1.82, *P* = 0.08) tests (Figure 1(a)). Both genotypes showed also the same latency for all measured behaviors in the hot plate test (Figure 1(b)): forepaw licking (*t*_49_ = 1.69, *P* = 0.10), hindpaw licking (*t*_49_ = 0.73, *P* = 0.47), and jump (*t*_48_ = 0.81, *P* = 0.42), suggesting that 5-HT_7_ receptor knockout mice perceive and respond normally to acute thermal nociceptive stimuli. In addition, formalin-induced licking and biting of the paw injected with formalin in 5-HT_7_ receptor knockout mice did not differ from wild-type mice. Repeated measures ANOVA (time × genotype) showed a significant effect of time (*F*_8,162_ = 24.30, *P* < 0.001), but no effect of genotype (*F*_1,162_ = 1.76, *P* = 0.19) and no interaction between these two factors (*F*_8,162_ = 0.90, *P* = 0.52). A slightly greater licking/biting time was observed 25 min after formalin injection in the 5-HT_7_ receptor knockout in comparison with wild-type mice, but difference was not significant (Figure 1(c)).

### 3.2. 5-HT_7_ Receptor Agonists Inhibited Selectively Phase II of Formalin-Induced Nociceptive Behavior in Wild-Type but Not in 5-HT_7_ Receptor Knockout Mice

To examine the *in vivo* functional specificity of 5-HT_7_ receptor agonists, AS-19, E-57431 and E-55888 were subcutaneously administered to wild-type and 5-HT_7_ receptor knockout mice, and treated animals were then subjected to nociceptive tests. Effective doses of AS-19 (10 mg/kg), E-57431 (10 mg/kg), and E-55888 (20 mg/kg) in reversing allodynia/hyperalgesia following capsaicin sensitization and nerve injury [13, 14] were used in these experiments. The 5-HT_7_ receptor agonists were administered in the formalin test at doses devoid of motor disturbing effects which could interfere with licking/biting behaviors, as previously described [14].

**Figure 2:**
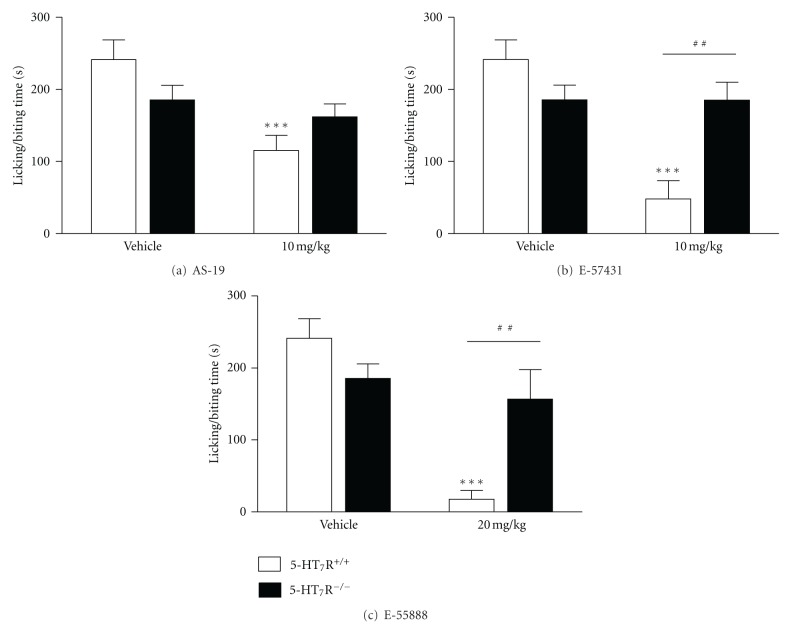
Effects of 5-HT_7_ receptor agonists AS-19 (a), E-57431 (b), and E-55888 (c) on formalin-induced nociceptive behaviors during phase II in wild-type and 5-HT_7_ receptor knockout mice. Subcutaneous administration of AS-19 (10 mg/kg), E-57431 (10 mg/kg), and E-55888 (20 mg/kg) significantly reduced the licking/biting time of the hind paw injected with formalin in wild-type but not in 5-HT_7_ receptor knockout mice. Each bar represents the mean ± S.E.M. (*n* = 7–12). ***P < 0.001 versus vehicle corresponding group; ^##^ *P* < 0.01 versus corresponding dose in 5-HT_7_ receptor knockout mice (Bonferroni multiple comparison test after ANOVA).

No significant effects were exerted by 5-HT_7_ receptor agonists on the response to thermal stimuli in the tail flick, tail immersion, and hot plate tests, and the formalin-induced phase I nociceptive behavior was not modified when 5-HT_7_ receptor agonists were administered to wild-type or 5-HT_7_ receptor knockout mice (data not shown). However, all three 5-HT_7_ receptor agonists inhibited phase II of the formalin-induced nociceptive behavior in wild-type mice, as evidenced by a reduction in the duration of licking/biting of the hindpaw injected with formalin (Figure 2). Two-way ANOVA (treatment × genotype) showed a significant effect of treatment after AS-19 administration (*F*_1,38_ = 10.34, *P* = 0.003), without genotype effect (*F*_1,38_ = 0.04, *P* = 0.84) and a significant interaction between these two factors (*F*_1,38_ = 4.86, *P* = 0.03). The comparison between treatments revealed a significant reduction of licking/biting time after AS-19 administration at 10 mg/kg in wild-type mice (*P* < 0.001; Figure 2). Two-way ANOVA calculated for E-57431 also showed a significant effect of treatment after E-57431 administration (*F*_1,39_ = 15.04, *P* < 0.001), without genotype effect (*F*_1,39_ = 2.64, *P* = 0.11) and a significant interaction between these two factors (*F*_1,39_ = 14.91, *P* < 0.001). The comparison between treatments revealed a significant reduction of licking/biting time after E-57431 administration at 10 mg/kg in wild-type mice (*P* < 0.001; Figure 2). In addition, a significant difference was found between genotypes when E-57431 was administered at 10 mg/kg (*P* < 0.01; Figure 2). In the same way, two-way ANOVA calculated for E-55888 also showed a significant effect of treatment after E-55888 administration (*F*_1,35_ = 22.3, *P* < 0.001), without genotype effect (*F*_1,35_ = 2.42, *P* = 0.13) and a significant interaction between these two factors (*F*_1,35_ = 13.32, *P* < 0.001). The comparison between treatments revealed a significant reduction of licking/biting time after E-55888 administration at 20 mg/kg in wild-type mice (*P* < 0.001; Figure 2). In addition, a significant difference was found between genotypes when E-55888 was administered at 20 mg/kg (*P* < 0.01; Figure 2). Interestingly, none of the three 5-HT_7_ receptor agonists exerted significant effects on formalin phase II nociceptive behavior in 5-HT_7_ receptor knockout mice (Figure 2).

### 3.3. Selective Doses of 5-HT_7_ Receptor Agonists Produced No Effect on Body Temperature in 5-HT_7_ Receptor Knockout and Wild-Type Mice

**Figure 3:**
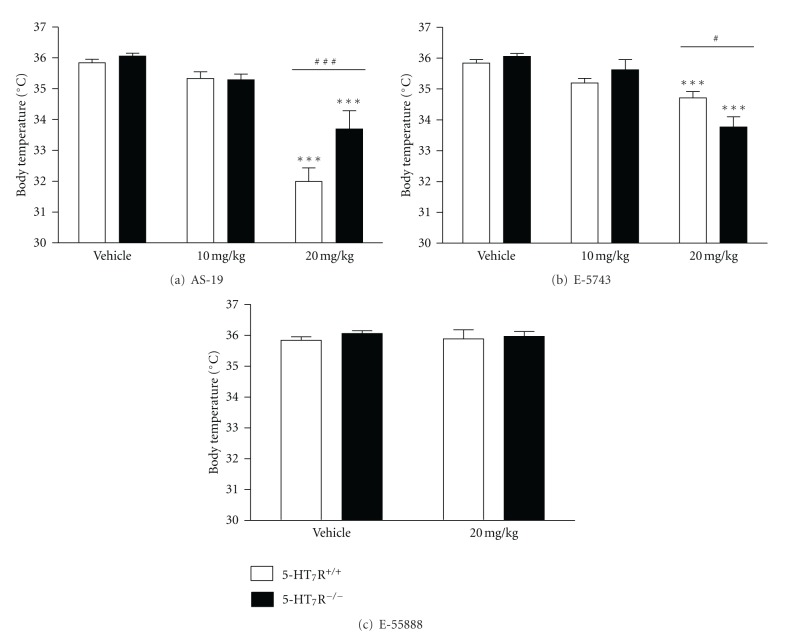
Effects of 5-HT_7_ receptor agonists AS-19 (a), E-57431 (b), and E-55888 (c) on body temperature in wild-type and 5-HT_7_ receptor knockout mice. Subcutaneous administration of AS-19 and E-57431 at 20 mg/kg significantly reduced body temperature in both wild-type and 5-HT_7_ receptor knockout mice and showed significant differences between both genotypes. However, E-55888 at 20 mg/kg did not reduce the body temperature neither in wild-type nor in 5-HT_7_ knockout mice. Each bar represents the mean ± S.E.M. (*n* = 8–12). ***P < 0.001 versus vehicle corresponding group; ^#^ *P* < 0.05; ^###^ *P* < 0.001 versus corresponding dose in 5-HT_7_ receptor knockout mice (Bonferroni multiple comparison test post-ANOVA).

The *in vivo* specificity of the 5-HT_7_ receptor agonists (AS-19, E-57431, and E-55888) was further examined using 5-HT_7_ receptor knockout and paired wild-type mice in the paradigm based on 5-HT_7_ receptor-mediated hypothermia [27]. Basal body temperature did not significantly differ in 5-HT_7_ receptor knockout and paired wild-type mice (36.1 ± 0.1°C and 35.8 ± 0.1°C, resp.; Figure 3). 

Two-way ANOVA (treatment × genotype) showed a significant effect of treatment after AS-19 administration (*F*_2,49_ = 62.17, *P* < 0.001), with genotype effect (*F*_1,49_ = 6.88, *P* = 0.01) and a significant interaction between these two factors (*F*_2,49_ = 4.77, *P* = 0.01). The comparison between treatments revealed a significant reduction of body temperature after AS-19 administration at 20 mg/kg in wild-type and 5-HT_7_ receptor knockout mice (*P* < 0.001; Figure 3). In addition, a significant difference was found between genotypes when AS-19 was administered at 20 mg/kg (*P* < 0.001; Figure 3). Two-way ANOVA calculated for E-57431 also showed a significant effect of treatment after E-57431 administration (*F*_2,49_ = 35.85, *P* < 0.001), without genotype effect (*F*_1,49_ = 0.31, *P* = 0.58) and a significant interaction between these two factors (*F*_2,49_ = 5.82, *P* = 0.005). The comparison between treatments revealed a significant reduction of body temperature after E-57431 administration at 20 mg/kg in wild-type and 5-HT_7_ receptor knockout mice (*P* < 0.001; Figure 3). In addition, a significant difference was found between genotypes when E-57431 was administered at 20 mg/kg (*P* < 0.05; Figure 3). However, two-way ANOVA calculated for E-55888 did not show significant differences on treatment after E-55888 administration (*F*_1,35_ = 0.02, *P* = 0.89), neither genotype effect (*F*_1,35_ = 0.91, *P* = 0.35) nor interaction between these two factors (*F*_1,35_ = 0.21, *P* = 0.65). The comparison between treatments and genotypes did not reveal significant reduction of body temperature after E-55888 administration at 20 mg/kg (*P* > 0.05; Figure 3).

Subcutaneous administration of doses of AS-19 (10 mg/kg), E-57431 (10 mg/kg), and E-55888 (20 mg/kg) which exerted analgesic effects in phase II formalin-induced pain, did not significantly change body temperature neither in 5-HT_7_ receptor wild-type nor in knockout mice (Figure 3). However, administration of a higher dose (20 mg/kg, s.c.) of the 5-HT_7_ receptor agonists AS-19 and E-57431 significantly reduced body temperature in both genotypes (Figure 3), suggesting that at such a high dose the selectivity window of AS-19 and E-57431 was overstepped. AS-19 at the 20 mg/kg dose produced a higher body temperature reduction in wild-type than in 5-HT_7_ receptor knockout mice (3.8 versus 2.4°C, resp.). In contrast, E-57431 at the same high dose (20 mg/kg) produced a lower body temperature reduction in wild-type than in 5-HT_7_ receptor knockout mice (1.1 versus 2.3°C, resp.) (Figure 3).

## 4. Discussion

In this study, the *in vivo* target-specific effects of the 5-HT_7_ receptor agonists AS-19, E-57431, and E-55888 on nociception (i.e., formalin-induced nociception) and thermoregulation were examined using 5-HT_7_ receptor knockout mice. These 5-HT_7_ receptor agonists exerted antinociceptive effects in phase II of the formalin test in wild-type but not in 5-HT_7_ receptor knockout mice, suggesting that their analgesic effect is actually 5-HT_7_ receptor mediated. Analgesic doses of 5-HT_7_ receptor agonists did not change body temperature neither in 5-HT_7_ receptor knockout nor in wild-type mice. However, a reduction in body temperature was observed in both genotypes when the dose of the agonists were increased up to levels exceeding their selectivity window.

The 5-HT_7_ receptor knockout mice offer a complementary approach to classical pharmacology and might provide insights into the functional implications of 5-HT_7_ receptors. To date, data obtained with these mutants suggest the involvement of 5-HT_7_ receptors in depression, schizophrenia, sleep, learning, locomotion, and hypothermia [25, 28, 34–37].

In this work, we demonstrated that sensitivity to noxious heat measured as the latency time of response to thermal stimulation in the tail flick, tail immersion, and hot plate tests did not differ in 5-HT_7_ receptor knockout compared to wild-type mice, as previously described [38]. In addition, the formalin-induced nociceptive behavior of 5-HT_7_ receptor knockout mice was not different from wild-type mice as no significant differences in licking/biting time were found between both genotypes, for either phase I or phase II of the formalin test. These results suggest that basic mechanisms for transduction, transmission, and perception of, as well as response to, nociceptive stimuli are intact in mice lacking 5-HT_7_ receptors. As previously reported, the loss of function of the missing 5-HT_7_ receptor could induce possible adaptive changes which could compensate for some alterations, thereby resulting in wild-type-like responses [38–40].

Thermal nociception and early phase response in the formalin test are caused predominantly by direct activation of peripheral C-fibers, whereas the late response (phase II) in the formalin test involves functional changes in the dorsal horn of the spinal cord (i.e., central sensitization) [41–43]. In this study, subcutaneous administration of 5-HT_7_ receptor agonists was devoid of activity in acute nociceptive tests (i.e., thermal- and formalin-induced phase I nociception), but exerted clear-cut antinociceptive effects in phase II of the formalin test in wild-type mice. These results are in line with previous studies showing that 5-HT_7_ receptor agonists and antagonists were ineffective in acute thermal nociceptive pain [44–47]. The lack of antinociceptive effects in thermal and phase I formalin-induced nociception, observed when 5-HT_7_ receptor agonists were administered, suggests no direct modulation by the 5-HT_7_ receptor subtype of acute nociceptive signals coming from small caliber unmyelinated nociceptive afferents. However, activation of spinal 5-HT_7_ receptors has been shown to play a role in the antinociceptive effects of opioids [44–47].

Our results on phase II formalin-induced behavior are in line with previous reports describing antinociceptive effects of selective 5-HT_7_ receptor agonists by systemic or spinal administration in neurogenic and neuropathic pain conditions involving central sensitization [13, 14, 17]. However, a clear-cut pronociceptive (proallodynic) effect was found when a 5-HT_7_ receptor agonist was administered intraplantarly into the ipsilateral hind paw injected with a low subactive dose of capsaicin [17]. In contrast, data in the literature using the formalin test suggest a pronociceptive role of both peripheral and spinal 5-HT_7_ receptors. Indeed, intraplantar or spinal administration of 5-carboxamidotryptamine, a nonselective 5-HT_7_ receptor agonist, increased phase II formalin-induced nociceptive behavior, and these effects were significantly reversed by the selective 5-HT_7_ receptor antagonist SB-269970 [23]. Differences could be due to species (mice versus rats), primary administration route (systemic versus spinal and local peripheral), selectivity of the 5-HT_7_ agonists used and animal models.

To further assess the *in vivo* specificity of the 5-HT_7_ receptor agonists used in this study, we examined in 5-HT_7_ receptor knockout mice, the effects of the 5-HT_7_ receptor agonists AS-19, E-57431, and E-55888 on both formalin-induced nociception and body temperature. Under our experimental conditions, 5-HT_7_ receptor agonists, at doses effective to reduce phase II formalin-induced nociceptive behavior, affected body temperature neither in wild-type mice nor in 5-HT_7_ knockout mutants. However, AS-19 and E-57431 at the high dose of 20 mg/kg significantly reduced body temperature not only in wild-type but also in 5-HT_7_ receptor knockout mice, indicating a non-5-HT_7_ receptor-mediated effect possibly due to interactions of these compounds with other 5-HT receptors when their selectivity window is surpassed. In line with this interpretation, we found that E-55888, the most selective 5-HT_7_ receptor agonist based on *in vitro* radioligand binding assays (Table 1), even at the dose of 20 mg/kg, did not exert any effect on body temperature in both genotypes. Taken together, the finding that the less selective agonists (AS-19 and E-57431) at high doses reduced body temperature in both wild-type and knockout mice, whereas the most selective one (E-55888) did not, suggests that activation of 5-HT_7_ receptors alone is not enough to affect body temperature. Our results do not rule out the possibility that 5-HT_7_ receptors might contribute to the regulation of body temperature by acting in concert with other serotonergic and/or nonserotonergic receptors. Indeed, we found a higher hypothermic effect induced by AS-19 (20 mg/kg) in wild-type compared to 5-HT_7_ receptor knockout mice, suggesting that 5-HT_7_ receptors might promote the decrease in body temperature when other mechanisms are also recruited. The *in vitro* binding profile of these ligands (see Table 1) suggests that 5-HT_1D_ and/or 5-HT_1A_ receptors could be involved in the observed hypothermic effects of AS-19 and E-57431, also because activation of these receptor types has been reported to induce hypothermia [16, 28, 48]. Overall, as previously reported, 5-HT_7_ receptors appear to be involved in a complex manner in thermoregulation, probably through mechanisms implicating direct/indirect interactions between 5-HT_7_ receptors and other molecular targets. 

## 5. Conclusions

Data obtained in this study strengthen the notion that 5-HT_7_ receptors play a role in nociceptive control in pain conditions involving central sensitization and add further support to their fine-tuning effects in body temperature homeostasis through possible actions in concert with other molecular targets. In addition, this study provides evidence that formalin-induced nociceptive behaviors and body temperature in 5-HT_7_ receptor knockout mice are useful models and relatively simple approaches to assess *in vivo* specificity of 5-HT_7_ receptor agonists.

## Figures and Tables

**Table 1:** Binding profiles of the 5-HT_7_ receptor agonists AS-19, E-57431, and E-55888.

Receptor	Affinity (K_*i*_ (nM))		
	AS-19	E-57431	E-55888
h5-HT_1A_	89.7 (149.5 x)	n.s.	700 (280 x)
r5-HT_1B_	490 (816.6 x)	n.s.	n.s.
h5-HT_1D_	6.6 (11 x)	53 (112.7 x)	n.s.
h5-HT_2A_	n.s.	560 (1191.5 x)	n.s.
h5-HT_2B_	n.s.	n.s.	n.s.
h5-HT_2C_	n.s.	n.s.	n.s.
h5-HT_3_	n.s.	n.s.	n.s.
h5-HT_4e_	—	n.s.	n.s.
gp5-HT_4_	n.s.	n.s.	—
h5-HT_5A_	98.5 (164.2 x)	n.s.	n.s.
h5-HT_6_	n.s.	n.s.	n.s.
h5-HT_7_	0.6	0.47	2.5
h5-HT transporter (SERT)	n.s.	n.s.	n.s.
Other receptors	n.s.^a^	n.s.^b^	n.s.^a^

n.s.: not significant (K_*i*_ > 1 *μ*M or less than 50% inhibition of specific radioligand binding at 1 *μ*M);

—: data not available.

gp: guinea pig; h: human; r: rat.

Data obtained from Brenchat et al. [13, 14]

Data in parentheses after K_*i*_ values represent the affinity ratio versus 5-HT_7_ receptors calculated as K_*i*_ for the tested receptor/K_*i*_ for 5-HT_7_ receptor. It is expressed as number-fold higher (x) for 5-HT_7_ than for the tested receptor.

^a^See the panel of other receptors assayed [13].

^b^See the panel of other receptors assayed [14].
